# Iatrogenic Aorto-Coronary Dissection Successfully Treated With IVUS Guided Unprotected Left Main Stenting: Case Report and Review of Literature

**DOI:** 10.14740/cr329w

**Published:** 2014-05-15

**Authors:** Avinash Murthy, Arti Singh, Edward R. Tuohy

**Affiliations:** a Division of Cardiology, Bridgeport Hospital, 267 Grant Street, Bridgeport, USA

**Keywords:** PCI, Aorto-coronary dissection, Left main coronary artery stenting, IVUS

## Abstract

A 52-year-old male underwent cardiac catheterization for abnormal stress test. Trans-radial coronary angiography revealed a severe proximal left anterior descending artery (LAD) lesion. LAD angioplasty was performed with two drug-eluting stents. This resulted in dissection of the proximal LAD, the circumflex artery and the left main coronary artery (LMCA) extending back into the coronary sinus. A diagnosis of type 3 coronary dissection was made. The circumflex artery and the left coronary artery were stented, and then the LMCA was stented. Repeat intravascular ultrasound showed resolution of the dissection and TIMI-3 flow was achieved in all vessels. He underwent follow-up angiography in 1 month, which revealed patent stents with resolution of the aorto-coronary dissection. We report a rare case of iatrogenic aorto-coronary dissection that was successfully treated with unprotected left main percutaneous coronary intervention strategy alone and review the pertinent literature.

## Introduction

Iatrogenic retrograde aorto-coronary dissection is a rare and potentially disastrous complication of percutaneous coronary intervention (PCI). The incidence of left main coronary artery (LMCA) dissection is reported at less than 0.1% in the published literature [1]. PCI leading to acute dissection of the ascending aorta has an incidence of about 0.02% to 0.1% of cases [2, 3]. LMCA dissection can lead to abrupt vessel closure with cessation of blood flow, subsequent pump failure and hemodynamic collapse. Before 1993, urgent coronary artery bypass grafting (CABG) was the treatment of choice, with many people dying before making it to the operating room. More recently, percutaneous bail-out LMCA stenting has been successfully performed [4].

## Case Report

**Figure 1:**
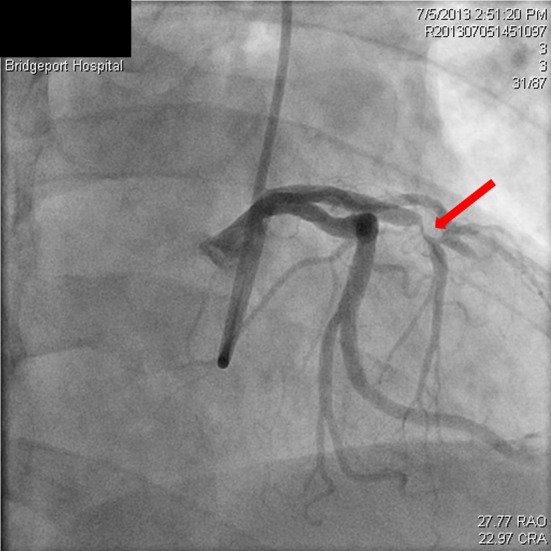
Subtotal occlusion of LAD.

**Figure 2:**
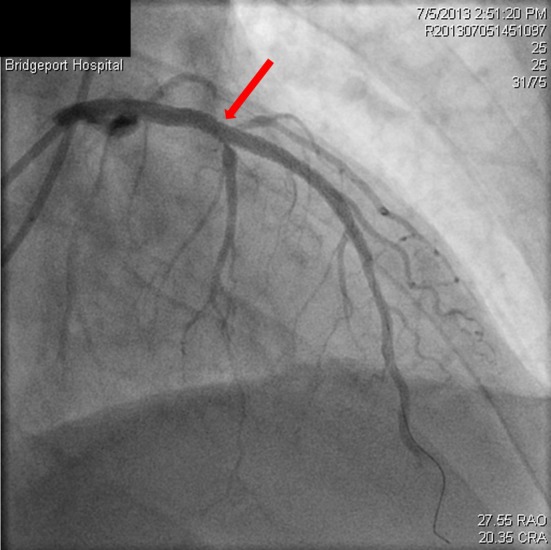
Successful stenting of LAD restores antegrade flow.

**Figure 3:**
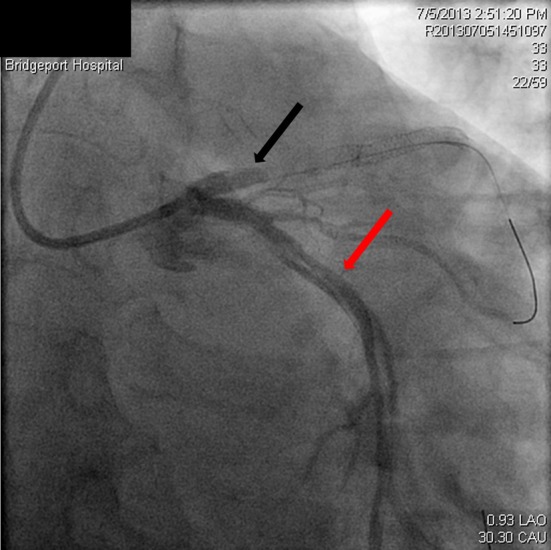
Spiral dissection of circumflex artery (red arrow), occluded LAD just prior to the stent (black arrow).

**Figure 4:**
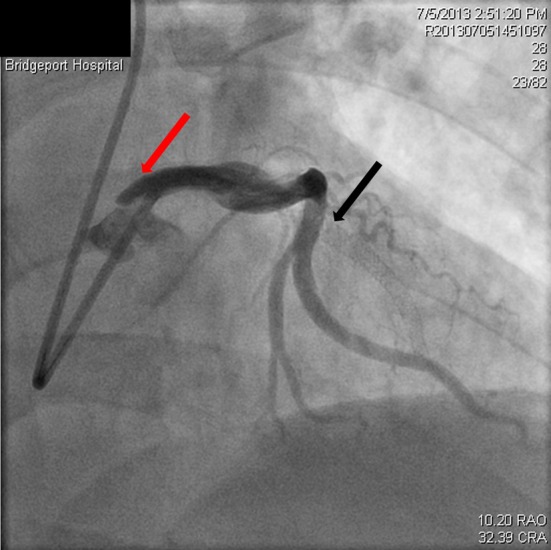
Dissection extending into the aorta (red arrow), occluded LAD (black arrow).

**Figure 5:**
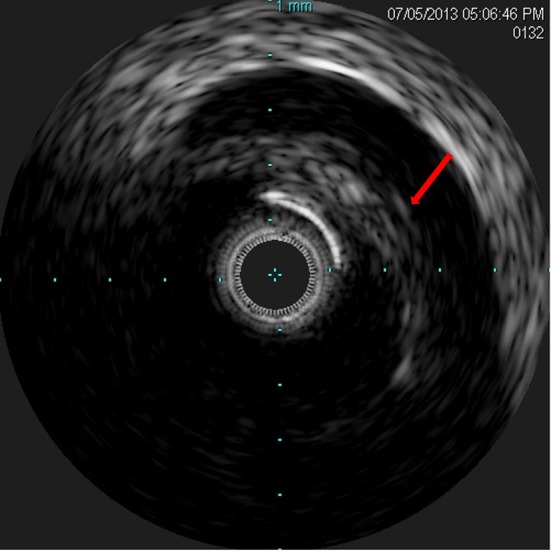
IVUS showing dissection flap in the LMCA.

**Figure 6:**
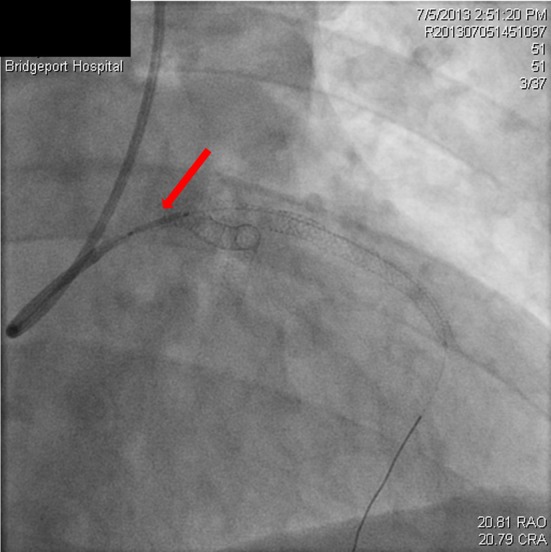
Stenting of the LMCA ostium.

**Figure 7:**
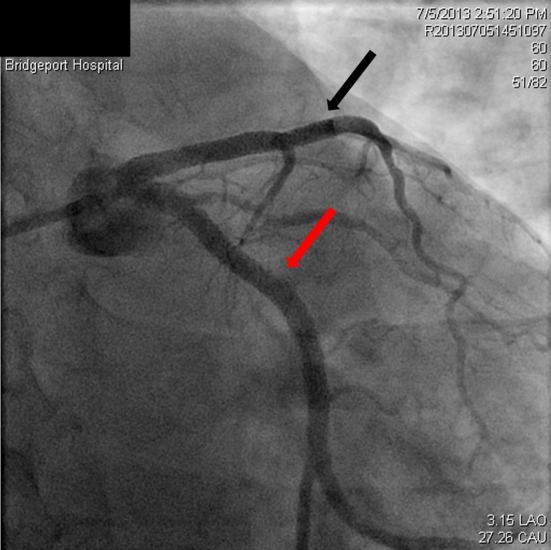
Restoration of flow in the circumflex artery (red arrow) and the LAD (black arrow).

A 52-year-old male with past medical history of hyperlipidemia and a remote 6-year smoking history presented with 2 weeks of chest pain and dyspnea on exertion. His family history was significant for myocardial infarction in his father at the age of 54 years. He was referred to the cardiac catheterization lab on an urgent basis from his cardiologist’s office, following a markedly abnormal stress MIBI test, which showed extensive anterior wall ischemia. Trans-radial coronary angiography revealed a subtotal occlusion proximal left anterior descending artery (LAD) lesion (Fig. 1). The rest of the coronary circulation was free of significant disease. LAD angioplasty was performed. Two drug-eluting stents (DESs) (PROMUS, 3.0/28 mm and 2.75/16 mm) were successfully implanted in the LAD from proximal to distal in an overlapping fashion with good result (Fig. 2). This restored the antegrade flow in the LAD. The more proximal LAD stent was then dilated with 3.5 mm balloon. On repeat angiography, it was evident that there was a dissection at the proximal edge of the LAD stent involving the LMCA extending back into the coronary sinus as well as spiraling down to the left circumflex coronary artery (LCx). A diagnosis of type 3 coronary dissection based on the simplified classification method was made (Fig. 3, 4) [1]. The arteries were rewired and accesses to the true lumen were gained. The LCx was stented with two DESs (PROMUS, 3.0/38 mm and 3.0/28 mm) back to its ostium. This completely restored flow to the LCx. Aspiration thrombectomy was performed in the proximal LAD and it was then stented with a DES (PROMUS, 3.0/28 mm) back into the left main artery. Intravascular ultrasound (IVUS) exam was performed and it revealed a dissection flap in the LMCA extending back into the coronary sinus involving the aorta (Fig. 5). The cardiothoracic surgery opinion was sought, but the patient refused bypass surgery, so we decided to stent the LMCA in an attempt to treat the aorto-coronary dissection. The LMCA was then stented with a DES (PROMUS, 4.0/18 mm) with about 2 mm extension into the aorta (Fig. 6). Repeat IVUS showed resolution of the dissection and final TIMI-3 flow was achieved in all vessels (Fig. 7). He underwent repeat angiography in 1 month, which revealed patent stents with resolution of the aorto-coronary dissection.

## Discussion

Iatrogenic coronary artery dissection results from various mechanisms including mechanical injury to the arterial wall due to extensive catheter manipulation, passage or deployment of an interventional device or inadvertent contrast administration when the tip of the catheter is scraping against the wall of the coronary artery during the injection. Many risk factors have also been identified including unusual/small coronary anatomy or location [3, 4], operator experience, presence of coronary atherosclerosis [5]. Older age, HTN, aortic root calcification and DM have been cited as clinical risk factors for coronary artery dissections [2]. Review of literature reveals that amplatz catheters are frequently a culprit with disproportionate increase in catheter-induced right coronary dissections [6]. Other catheters including left Judkins shape guiding catheters (Amplatzer left, EBU or Q-curve), brachial approach with Castillo or Sones catheters have also been implicated [1, 6]. Deep engagement of guiding catheters (especially 7 Fr and 8 Fr sizes) and use of stiff wires have been associated with dissections [5]. In settings of ACS/acute myocardial infarction, pro-inflammatory marker and pro-apoptotic protein expression (cathepsin B) surge can further predispose patients to dissection [7].

Aorto-coronary dissection should be suspected in any patient undergoing a percutaneous coronary artery intervention who develops otherwise unexplained hypotension and hemodynamic compromise. Once dissection occurs, the clinical picture differs depending on the remaining antegrade coronary flow, and can range from an asymptomatic patient with preserved TIMI-3 flow, to a patient in refractory cardiogenic shock whose LMCA is completely compromised. While iatrogenic aorto-coronary artery dissection is obvious on coronary angiography, it may under estimate the extent of aortic involvement. Physical exam findings such as a murmur of aortic regurgitation and diminished peripheral pulses are less prevalent. Even in cases of initially preserved TIMI-3 flow with stable hemodynamics, deterioration may acutely occur because of compromise to flow from a progressive dissection or a superimposed thrombus formation [4]. Hence, every dissection mandates immediate formulation of a good management plan.

Coronary dissections are classified using the National Heart, Lung, and Blood Institute (NHLBI) diagnostic criteria-NHLBI. The NHLBI has six types of dissections classified based on the extent and location of radiolucent areas within the coronary lumen during contrast injection and persistence of contrast. The simplified classification scheme defines three types of coronary dissections involving the left main artery. Type 1 is a localized dissection without extension into the LAD or LCx. Extension of LMCA dissection into LAD or LCx is defined as type 2, and if extension involves the aortic root then it is type 3 [1].

Importance of prompt and timely recognition of this complication to prevent both retrograde and antegrade extension cannot be overemphasized. Conservative therapy, bailout stent implantation and emergent CABG have all been described extensively in literature as potential strategies for the management of iatrogenic aorto-coronary dissection. Given the unpredictable nature of the dissection and the potential for catastrophic sequelae, conservative therapy is rarely considered as a therapeutic option for aorto-coronary dissection. In the setting of aorto-coronary dissection and hemodynamic collapse, IABP alone will not provide hemodynamic stability [6, 8], and extracorporeal support is not readily available in all centers. Successful treatments (namely percutaneous or surgical) of any arterial dissection requires sealing of entry site to thrombose the false lumen and thus prevent progression of vessel dilatation and/or rupture [9]. Some operators have suggested that for dissections starting downstream and propagating retrograde, the site of initial coronary dissection should be stented first with retrograde stenting of the aorta if needed. While others have proposed immediate ostial stenting irrespective of whether the dissection starts from the ostium or further downstream in the coronary [9].

Dunning et al [10] have suggested that patients be managed by surgery if the dissection extends beyond 40 mm from the coronary ostium. However, Carstensen et al [9], based on their study, suggest immediate ostial stenting even when significant aortic involvement has occurred, as the ostial stent has high changes of successfully sealing the dissection and it does not compromise the chance of a future surgical success. Among the 36 patients studied by Cheng et al [11] with back-up CABG strategy for failed bail-out-stenting in aorto-coronary dissections an overall 94.4% survival was noted.

The stent must be delivered to the coronary ostium, protruding 1 - 2 mm into the aortic lumen, with the intention of sealing the aortic dissection. The flaring of the proximal end of the stent should be done to avoid in-stent restenosis, and to facilitate later catheter engagements. Intracoronary stenting of the LAD and LCx can be accomplished using various bifurcation stenting techniques. Literature review cites that mostly DES has been used although bare metal stents have also been used in iatrogenic aorto-coronary dissection treated with LMCA stenting. Initial LMCA stenting experiences were described by Hokken et al in 2002 [12], where 18 patients out of 7,199 found to have LMCA dissection were treated with stenting. NIR stents were used in six patients, BX velocity stents in four patients, and multilink stent in one patient and Jomed bifurcation stent on one patient. In six patients the stent type was not recorded. About 56% (10/18) patients required no surgical intervention and were free of cardiac complications 3 years status-post stenting.

Surgical repair, given the setting of full anticoagulation/antiplatelet therapy and acute coronary ischemia, might have a higher overall risk than bail-out-stenting [9]. In institutions without surgical back-up, CABG as a primary management strategy may result in increased rate of MI and death related to delay in time to surgery and prolonged ischemia during the waiting period [1]. Stenting does not appear to compromise chances of a successful surgical intervention should the initial PCI approach fail [9]. We think surgery should only be considered on the occasion when stenting fails to seal the dissection or if the aortic valve becomes involved. Alternatively, in patients with dissection induced aortic regurgitation or hemopericardium, immediate surgical intervention should be sought.

Problems do remain with LMCA stenting, as stated in the study by Onsea et al [4], uncontrollable bleeding, early stent thrombosis, interruption of dual antiplatelet therapy are encountered and the consequences can be devastating. The study by Eshtehardi et al [1] has suggested there is no difference in the in-hospital outcome based on the initial revascularization strategy. Five-year follow-up outcomes also did not reveal any statistically significant differences between surgical revascularization and left main stenting. In the initial PCI strategy group, in-stent restenosis, particularly distal to the stented site has been reported necessitating bypass surgery [5]. In the study by Cheng et al [11] the binary stenosis rate of the LMCA was around 30% at 5 ± 2 months follow-up with no difference in the long-term outcome when compared to elective PCI of unprotected LMCA. Follow-up angiography has been done to assess these patients. CT is a great non-invasive means to document healing and to check patency of the stents in these patients, the need to use contrast renders it a less desirable option than transesophageal echocardiography.

Our patient was discharged home the day after the procedure without any complications. The patient underwent surveillance angiography in 1 month from initial cardiac catheterization date. This was technically not difficult and showed full patency of original stents without any extension of the dissection plane. We report a rare case of iatrogenic aorto-coronary dissection that was successfully treated with PCI strategy alone.
